# *β*-Enamino Esters in Heterocyclic Synthesis: Synthesis of Pyrazolone and Pyridinone Derivatives

**DOI:** 10.3390/molecules15064359

**Published:** 2010-06-15

**Authors:** Abdellatif Mohamed Salaheldin, Mariam Abdullah Al-Sheikh

**Affiliations:** 1 Department of Chemistry, Faculty of Science, Cairo University, Giza-12613, Egypt; 2 Department of Chemistry, Girls College of Education, Jeddah, P. O. Box 138016, Jeddah 21323, Saudi Arabia

**Keywords:** *β*-enamino esters, pyrazolone, pyridinone, phenylhydrazones

## Abstract

An efficient and convenient synthesis of pyrrolidinones and pyridinones utilizing enamino esters as starting material has been described. The structures of the compounds obtained were confirmed by spectral and elemental analyses.

## 1. Introduction

*β*-Enamino esters are versatile intermediates for the synthesis of nitrogen containing compounds [1,2,3,4,5,6,7,8]. Also, they are important subunits present in some biologically important natural products as well as therapeutic agents [9,10,11,12]. Due to the importance of *β*-enamino ester derivatives as bioactive leads and versatile building blocks, their synthesis and applications have long been an active topic in organic synthesis [13,14,15,16,17,18,19]. 

As part of our ongoing studies on the synthesis of nitrogen-containing compounds and in conjunction of our interest in the chemistry of enamines [20,21,22,23,24,25], we report herein the synthesis of the starting enamino esters **2** and **3** and a study of their reactivity towards some selected nitrogen and carbon nucleophiles as well as benzenediazonium salts to synthesize the new pyrazolone, pyridinone and phenylhydrazone derivatives with the expectation that they would be of biological interest. In our chemical reactivity studies described here, we principally employed the intermediates **2b** and **3** due to their easy preparation and good yield of the subsequent reactions.

## 2. Results and Discussion

**Scheme 1:**
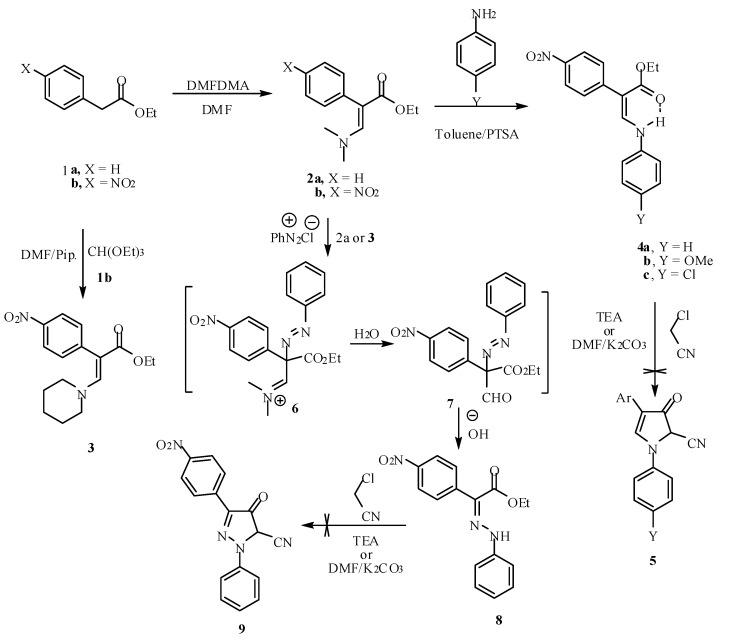
Synthesis and reactivity of *β*-enamino esters.

Reaction of ethyl phenyl acetate and ethyl *p*-nitrophenyl acetate **1a,b** with *N,N*-dimethylformamide dimethyl acetal (DMFDMA) in DMF at 60 °C for 4h yielded the enamino esters **2a,b** in good yields. On the other hand, the enamino ester **3** was prepared by reacting compound **1b** with triethyl orthoformate and piperidine in DMF at reflux temperature for 24 h (Scheme 1). The structures of the enamino esters **2a,b** and **3** were confirmed by mass spectrometry, ^1^H- and ^13^C-NMR. For example, the ^1^H-NMR spectrum of compound **3** showed two broad signals for the piperidinyl protons at δ = 1.48 (3 CH_2_) and 3.01 (2 CH_2_) ppm and singlet signal at δ = 7.63 ppm for the olefinic proton, besides the signals of ester and aromatic protons in their expected positions (see Experimental).

The reaction of enamino esters **2b** or **3** with aromatic amines in refluxing toluene and in the presence of *p*-toluenesulfonic acid (PTSA) afforded compounds **4a-c** as the only reaction products. Attempts to convert compounds **4** into the corresponding pyrrolidinone derivatives **5** either by heating with chloroacetonitrile in triethylamine or in DMF/K_2_CO_3_ were unsuccessful (Scheme 1) [25,26].

Coupling compound **2b** or **3** with benzenediazonium chloride furnished the phenylhydrazone **8**. It is believed that nitrogen lone pair resonance increases the nucleophility of C-2 and that the diazonium salts **6,** which are initially formed, are hydrolyzed under the reaction conditions and then underwent a Japp-Klingmann type of cleavage to yield **8,** which could not be obtained by direct coupling withbenzenediazonium chloride. Compound **8** failed to react with chloroacetonitrile in triethylamine or in DMF/K_2_CO_3_ to give the pyrazolone **9** (Scheme 1). 

**Scheme 2:**
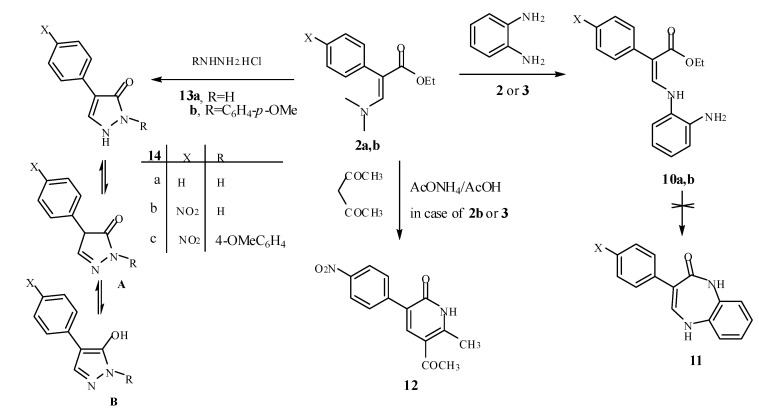
Reaction of *β*-enamino ester with hydrazines, amine and active methylen group.

We envisaged that the reaction of **2a,b** with *o*-phenylenediamine in refluxing toluene and in the presence of PTSA might afford the 2-phenylacrylate derivatives **10a,b** or diazepene derivative **11** (Scheme 2). The identity of compounds **10** was supported by a correct element analysis and spectral data. Thus, IR spectrum of **10b** relived a broad absorption bands at 3,448 (NH), 3,325 (NH_2_) cm^-1^, corresponding to NH and NH_2_ stretching, and 1,664 for C=O absorption. The ^1^H-NMR spectrum displayed the presence of broad signals at δ = 3.65 ppm and a doublet signal at δ = 10.41 ppm with *J* coupling = 12.6 Hz, (D_2_O exchanged for both signals), assignable to a NH_2_ group and the NH. A doublet signal at δ = 7.45 ppm with *J* coupling = 12.6 Hz, was assigned for H-3 (=CH), besides signals due to the ester group and aromatic protons in their expected positions. Additionally, its structure was fully confirmed by ^13^C-NMR, which was compatible with the suggested structure. Furthermore, in its mass spectrum, this product has the molecular ion *m/z* = 327 (84%), also confirming its presumed structure. Analytical data are thus all in accordance with the proposed structure for compound **10b**. Our efforts to synthesize the interesting diazepenes **11** *via* the intramoleculer condensation of compound **10** failed (Scheme 2) [27,28].

Next, we investigated the reaction of compounds **2a,b** or **3** with acetylacetone in acetic acid in the presence of ammonium acetate that afforded the pyridinone **12**. This find is similar to the reported synthesis of pyridines from the reaction of enaminones with acetylacetone under similar conditions [29]. On the other hand, when compounds **2b** or **3** reacted with substituted hydrazines **13a,b** in ethanol at reflux temperature for 3h they afforded the pyrazolone derivatives **14a-c** [30]. Although pyrazolone derivatives **14** can also exist as **14A** or hydroxypyrroles **14B**, the pyrazolone structure **14** is established based on the presence of carbonyl absorption band in IR spectra and also the ^1^H-NMR spectra that revealed pyrazolone H-5 and NH signals. Moreover, structure **14** was confirmed by the ^13^C-NMR spectra which allowed an unambiguous assignment in the ^1^H- and ^13^C-NMR spectra (see Experimental). 

## 3. Experimental

### 3.1. General

The melting points were determined on a Stuart melting point apparatus. The IR spectra were recorded as KBr pellets using a FTIR Bruker-Vector 22 spectrophotometer. The ^1^H and ^13^C-NMR spectra were recorded in DMSO-d_6_ or CDCl_3_ as solvent, on a Varian Gemini 300 MHz NMR spectrometer using TMS as internal standard. Double resonance, HMQC and HMBC experiments were carried out for complete assignment of ^1^H and ^13^C peaks in the NMR spectra, whenever possible. Chemical shifts are reported in δ units (ppm). Mass spectra were measured on a Shimadzu GCMS-QP-1000 EX mass spectrometer in EI (70 ev) mode. The elemental analyses were performed at the Micro analytical Center, Cairo University, Egypt.

### 3.2. Synthesis of ethyl 3-(dimethylamino)-2-arylacrylates ***2a,b***

To a solution of ethyl phenylacetate **1a** or ethyl 4-nitrophenylacetate **1b** (0.01 mol) in DMF (10 mL), DMFDMA (0.012 mol) was added. Then, the mixture was heated at 60 °C for 4 h. After cooling to r.t., the mixture was left standing overnight, then the resulting solid product was collected by filtration and washed with ethanol to give **2b**. In case of compound **2a**, brine (10 mL) was added to the reaction mixture. After extraction with CH_2_Cl_2_ (3 × 10 mL), the combined organic fractions were dried over MgSO_4_ and concentrated under vacuum to afford compound **2a** as a brown oil.

*Ethyl 3-(dimethylamino)-2-phenylacrylate* (**2a**). Yield 86%, ^1^H-NMR (CDCl_3_): δ = 1.18 (t, 3H, *J* = 7.5 Hz, CH_3_), 2.65 (s, 6H, 2CH_3_), 4.10 (q, 2H, *J* = 7.5 Hz, CH_2_), 7.17–7.29 (m, 5H, Ar-H), 7.57 (s, 1H, C*H*-olfeinic-H-3), MS (EI, 70eV): *m/z* = 219 (M^+^); Anal. Calcd. for C_13_H_17_NO_2_ (219.13): C, 71.21; H, 7.81; N, 6.39. Found: C, 71.34; H, 7.69; N, 6.52.

*Ethyl 3-(dimethylamino)-2-(4-nitrophenyl)acrylate* (**2b**). Orange crystals (85%), mp 127–129 °C; IR (KBr) ν = 1,668 (C=O), 1,463, 1,376 (NO_2_) cm^-1^; ^1^H NMR (CDCl_3_): δ = 1.20 (t, 3H, *J* = 7.5 Hz, CH_3_), 2.73 (s, 6H, 2CH_3_), 4.14 (q, 2H, *J* = 7.5 Hz, CH_2_), 7.32 (d, 2H, *J* = 9.0 Hz, H-2´,6´), 7.65 (s, 1H, C*H*-olfeinic- H-3), 8.14 (d, 2H, *J* = 9.0 Hz, H-3´,5´); ^13^C-NMR (CDCl_3_): δ = 14.46 (CH_3_), 43.64 (2CH_3_), 59.80 (CH_2_), 97.27 (*C*=CH,C-2), 122.36 (C-3´,5´), 132.39 (C-2´,6´), 144.45 (C-1´), 145.75 (C-4´), 150.50 (C=*C*H,C-3), 168.73 (CO). MS (EI, 70eV): *m/z* = 264 (M^+^). Anal.Calcd. for C_13_H_16_N_2_O_4_ (264.28) :C, 59.08; H, 6.10; N, 10.60. Found: C, 58.87; H, 6.21 ; N, 10.68. 

### 3.3. Synthesis of ethyl 2-(4-nitrophenyl)-3-(piperidin-1-yl)acrylate ***(3)***

A mixture of ethyl 4-nitrophenylacetate **1b** (0.2 mol), triethylorthoformate (0.2 mol) and piperidine (0.2 mol) in DMF (30 mL) was refluxed for 24 h. The reaction mixture was then cooled to r. t. and poured into water. The solid product thus formed was collected by filtration and recrystallized from ethanol. m.p. 94–95 °C. Yield: 75%. IR (KBr) ν= 1,665 (C=O), 1,463, 1,377 (NO_2_) cm^-1^; ^1^H-NMR (CDCl_3_): δ = 1.19 (t, 3H, *J* = 7.5 Hz, CH_3_), 1.47–1.52 (m, 6H, 3CH_2_), 2.98–3.03 (m, 4H, 2CH_2_), 4.13 (q, 2H, *J* =7.5 Hz, CH_2_), 7.33 (d, 2H, *J* = 9Hz, H-2´,6´), 7.63 (s, 1H, C*H*-olfeinic- H-3), 8.15 (d, 2H, *J* = 9 Hz, H-3´,5´); ^13^C-NMR (CDCl_3_): δ = 14.46 (CH_3_), 23.53 (2CH_2_), 25.62 (2CH_2_), 52.16 (CH_2_), 59.75 (OCH_2_), 96.15 (*C*=CH, C-2), 122.74 (C-3´,5´), 131.93 (C-2´,6´), 145.14 (C-1´), 145.80 (C-4'), 149.39 (C=*C*H,C-3), 168.89 (CO); MS (EI, 70 eV): *m/z* = 304 (M^+^); Anal. Calcd. for C_16_H_20_N_2_O_4_ (304.34) :C, 63.14; H, 6.62; N, 9.20. Found: C, 62.99; H, 6.80; N, 8.99. 

### 3.4. General procedure for preparation of 3-arylamino-2-(4-nitrophenyl)acrylate derivatives ***4a**-**c*** and ***10a,b***

Aromatic amines (0.01 mol) and *p*-toluenesulfonic acid (0.15 g) were added to a solution of enamino esters **2a,b** or **3** (0.01 mol) in toluene (25 mL). The reaction mixture was refluxed for 7 h. After cooling to r.t., the precipitated solid product was collected by filtration and recrystallized from the proper solvents to afford **4a-c** and **10a,b**, respectively.

*Ethyl-2-(4-nitrophenyl)-3-(phenylamino)acrylate* (**4a**). Colorless needles, 75% yield, mp 140–141 °C; IR (KBr) ν = 3,448 (NH), 1,664.9 (C=O), 1,460, 1,370 (NO_2_) cm^-1^; ^1^H- NMR (CDCl_3_): δ= 1.33 (t, 3H, *J* = 7.5 Hz, CH_3_), 4.29 (q, 2H, *J* = 7.5 Hz, CH_2_); 7.06-7.11 (m, 3H, H-4´´, 3´´,5´´); 7.33-7.38 (m, 2H, H-2´´, 6´´), 7.51 (d, 2H, *J* = 9.2 Hz, H-2´,6´), 7.55 (d, 1H, *J* = 12.8 Hz, H-3), 8.19 (d, 2H, *J* = 9Hz, H-3´,5´), 10.57 (d, 1H, *J* = 12.8 Hz, NH); ^13^C NMR (CDCl_3_): δ = 14.32 (CH_3_), 60.31 (CH_2_), 101.17 (C-2), 116.13 (C-2´´, 6´´), 123.38 (C-3´, 5´), 123.71 (C-4´´), 129.38 (C-2´, 6´), 129.82 (C-3´´, 5´´), 140.05 (C-1´´), 144.77 (C-1´), 145.23 (C-3), 145.65 (C-4´), 169.36 (CO); MS (EI, 70eV): *m/z* = 312 (M^+^); Anal. Calcd. for C_17_H_16_N_2_O_4_ (312.32): C, 65.38; H, 5.16; N, 8.97. Found: C, 65.52; H, 5.04; N, 8.90 %.

*Ethyl-3-(4-methoxyphenylamino)-2-(4-nitrophenyl) acrylate* (**4b**). Colorless needles, 75% yield, mp 102–104 °C; IR (KBr) ν= 3,446 (NH), 1,665 (C=O), 1,469, 1,372 (NO_2_) cm^-1^; ^1^H-NMR (CDCl_3_): δ= 1.33 (t, 3H, *J* = 7.5 Hz, CH_3_), 3.81 (s, 3H, OCH_3_), 4.28 (q, 2H, *J* = 7.5 Hz, CH_2_); 6.88 (d, 2H, *J* = 9.0 Hz, H-3´´,5´´); 7.03 (d, 2H, *J* = 9.0 Hz, H-2´´, 6´´), 7.43 (d, 1H, *J* = 12.9 Hz, H-3), 7.51 (d, 2H, *J* = 9.0 Hz, H-2´,6´), 8.18 (d, 2H, *J* = 9.0 Hz, H-3´,5´), 10.52 (d, 1H, *J* = 12.9 Hz, NH); ^13^C-NMR (CDCl_3_): δ = 14.35 (CH_3_), 55.54 (OCH_3_), 60.16 (CH_2_), 99.11 (C-2), 116.44 (C-3´´, 5´´), 118.80 (C-2´´, 6´´), 124.73 (C-3´, 5´), 130.37 (C-2´, 6´), 135.60 (C-1´´), 146.54 (C-1´), 147.77 (C-4´), 148.26 (C-3), 157.01 (C-4´´), 170.75 (CO); MS (EI, 70eV): *m/z* = 342 (M^+^); Anal. Calcd. for C_18_H_18_N_2_O_5_ (342.35): C, 63.15; H, 5.30; N, 8.18. Found: C, 62.99; H, 5.11; N, 8.31 %.

*Ethyl-3-(4-chlorophenylamino)-2-(4-nitrophenyl) acrylate* (**4c**). Yellow crystals, 82% yield, mp 119–120 °C; IR (KBr) ν= 3,447 (NH), 1,665 (C=O), 1,459, 1,371 (NO_2_) cm^‑1^; ^1^H-NMR (DMSO-d_6_): δ= 1.24 (t, 3H, *J* = 7.5 Hz, CH_3_), 4.22 (q, 2H, *J* = 7.5 Hz, CH_2_); 7.26 (d, 2H, *J* = 8.8 Hz, H-2´´,6´´), 7.55 (d, 2H, *J* = 8.8 Hz, H-3´´,5´´), 7.68 (d, 2H, *J* = 8.8 Hz, H-2´,6´), 7.86 (d, 1H, *J* = 12.8 Hz, H-3), 8.16 (d, 2H, *J* = 9Hz, H-3´,5´), 10.49 (d, 1H, *J* = 12.8 Hz, NH); ^13^C-NMR (DMSO-d_6_): δ = 14.75 (CH_3_), 60.44 (CH_2_), 101.32 (C-2), 118.38 (C-2´´, 6´´), 123.49 (C-3´, 5´), 129.77 (C-3´´, 5´´), 130.29 (C-2´, 6´), 132.56 (C-4´´), 139.78 (C-1´´), 141.80 (C-1´), 144.20 (C-4´), 145.73 (C-3), 167.89 (CO); MS (EI, 70 eV): *m/z* = 346 (M^+^, ^35^Cl). 348 (M^+^, ^37^Cl); Anal. Calcd. for C_17_H_15_ClN_2_O_4_ (346.76): C, 58.88; H, 4.36; N, 8.08. Found: C, 58.59; H, 4.18; N, 8.14%.

*Ethyl 3-(2-aminophenylamino)-2-phenylacrylate* (**10a**). Colorless needles, 75% yield, mp 156–158 °C; IR (KBr) ν = 3,440 (NH), 3,317 (NH_2_), 1,670 (C=O) cm^-1^; ^1^H-NMR (DMSO‑d_6_): δ= 1.37 (t, 3H, *J* = 7.5 Hz, CH_3_), 3.80 (brs, 2H, NH_2_), 4.27 (q, 2H, *J* = 7.5 Hz, CH_2_); 6.90-6.96 (m, 2H, Ar-H), 6.98–7.06 (m, 2H, Ar-H), 7.14–7.22 (m, 5H, Ar-H), 7.42 (d, 1H, *J* = 12.6 Hz, H-3), 10.41 (d, 1H, *J* = 12.6 Hz, NH); MS (EI, 70 eV): *m/z* = 282 (M^+^); Anal. Calcd. for C_17_H_18_N_2_O_2_ (282.34): C, 72.32; H, 6.43; N, 9.92;. Found: C, 72.26; H, 6.79; N, 10.22.

*Ethyl-3-(2-aminophenylamino)-2-(4-nitrophenyl) acrylate* (**10b**). Colorless needles, 75% yield, mp 186–188 °C; IR (KBr) ν = 3,448 (NH), 3,325 (NH_2_), 1,664 (C=O), 1,465, 1,378 (NO_2_) cm^-1^; ^1^H-NMR (DMSO‑d_6_): δ= 1.33 (t, 3H, *J* = 7.5 Hz, CH_3_), 3.65 (brs, 2H, NH_2_), 4.32 (q, 2H, *J* = 7.5 Hz, CH_2_); 6.82-6.88 (m, 2H, Ar-H), 6.98–7.06 (m, 2H, Ar-H), 7.45 (d, 1H, *J* = 12.6 Hz, H-3),, 7.50 (d, 2H, *J* = 9.0 Hz, H-2´,6´), 8.17 (d, 2H, *J* = 9.0 Hz, H-3´,5´), 10.36 (d, 1H, *J* = 12.6 Hz, NH); ^13^C-NMR (DMSO-d_6_): δ = 14.49 (CH_3_), 59.77 (CH_2_), 96.86 (C-2), 115.07 (C-3"), 121.29 (C-4"), 122.50 (C-3´, 5´), 122.74 (C-5"), 123.17 (C-6''), 132.06 (C-1"), 132.46 (C-2´, 6´), 136.04 (C-2"), 145.20 (C-1´), 145.97 (C-4´), 149.40 (C-3), 169.70 (CO); MS (EI, 70 eV): *m/z* = 327 (M^+^); Anal. Calcd. for C_17_H_17_N_3_O_4_ (327.33): C, 62.38; H, 5.23; N, 12.84. Found: C, 62.26; H, 5.08; N, 12.77.

### 3.5. General procedure for preparation of ethyl (4-nitrophenyl)phenylhydrazono acetate *(**8**)*

A solution of benzenediazonium chloride salt (10 mmol), prepared by adding sodium nitrite solution (0.7 g in 10 mL of H_2_O) to a chilled solution of aniline hydrochloride (10 mmol of aniline in5 mL of conc. HC1) with stirring, was added to a cold solution of ethyl 3-substituted-2-(4-nitrophenyl)acrylates **2b** or **3** in ethanol (50 mL) containing sodium acetate (10 mmol). The reaction mixture was stirred for 1 h. The solid product formed was collected by filtration, washed well with water and recrystallized from ethanol. The title compound was obtained in 90% yield as yellow crystals, mp 145–146 °C, IR (KBr) v= 3,220 (NH); 1,669 (C=O) cmˉ^1^; ^1^H-NMR (DMSO-d_6_): δ=1.16 (t, 3H, *J* = 7.5 Hz, CH_3_), 4.25 (q, 2H, *J* = 7.5 Hz, CH_2_); 7.11–7.25 (m, 3H, Ar-H), 7.30–7.40 (m, 4H, Ar-H); 7.62 (d, 2H, *J* = 8.5 Hz, Ar-H), 11.78 (bs, 1H, NH), MS (EI, 70eV): *m/z* = 313 (M^+^); Anal. Calcd. for C_16_H_15_N_3_O_4_ (313.31): C, 61.34; H, 4.83; N, 13.41. Found: C, 61.42; H, 4.88; N, 13.38 %.

### 3.6. Synthesis of 5-acetyl-6-methyl-3-(4-nitrophenyl)pyridin-2(1H)-one *(**12**)*

To a mixture of enamino ester **2b** or **3** (4 mmol) and the acetylacetone (4 mmol) in acetic acid (10 mL), ammonium acetate (6 mmol) was added, then the reaction mixture was refluxed for 6 h. After cooling to r.t., the precipitated solid product was collected by filtration and recrystallized from DMF/EtOH (1:3) to give compound **12** as brown crystals, yield (70%), mp 260–262 °C; IR (KBr) ν = 3,225 (NH); 1,680 (C=O); 1,660 (C=O), 1,469, 1,374 (NO_2_) cm^-1^; ^1^H-NMR (DMSO-d_6_): δ = 2.21 (s, 3H, CH_3_), 2.65 (s, 3H, CH_3_), 7.28 (d, 2H, *J* = 8.8 Hz, Ar-H), 7.79–7.88 (m, 3H, Ar-H), 8.01 (s, 1H, NH); MS (EI, 70 eV): *m/z* = 272 (M^+^); Anal. Calcd. for C_14_H_12_N_2_O_4_ (272.26): C, 61.76; H, 4.44; N, 10.29. Found: C, 61.81; H, 4.32; N, 10.35 %.

### 3.7. General procedure for preparation of 4-aryl pyrazolo-3-one *(**14**)*

A mixture of enamino esters **2a,b** or **3** (0.01 mol) and substituted hydrazine hydrochlorides **13a,b** (0.01 mol) in ethanol (25 mL) was refluxed for 3h. After cooling to r.t., the reaction mixture was poured into cold water. The resulting solid was collected by filtration and washed with ethanol.

*4-Phenyl-1,2-dihydropyrazol-3-one* (**14a**). Orange crystals, 80% yield, mp 199–200 °C; IR (KBr) ν = 3,430 (NH), 1,666 (C=O) cmˉ^1^; ^1^H-NMR (DMSO-d_6_): δ = 7.06 (t, 1H, *J* = 8.2 Hz, H-4´), 7.29 (t, 2H, *J* = 8.2 Hz, H-3´, 5´), 7.66 (d, 2H, *J* = 8.2 Hz, H-2´, 6´), 7.88 (s, 1H, H-5), 10.76 (bs, 1H, NH), 11.14 (br s, 1H, NH); ^13^C-NMR (DMSO-d_6_): δ = 104.68 (C-4), 125.22 (C-4´), 125.49 (C-3´, 5´), 127.94 (C-5), 128.89 (C-2´, 6´), 133.83 (C-1´), 158.94 (C=O); MS (EI, 70 eV): *m/z* = 160 (M^+^); Anal. Calcd. for C_9_H_8_N_2_O (160.17): C,67.49; H,5.03; N,17.49. Found:C,67.52; H,4.98; N, 17.60. 

*4-(4-Nitrophenyl)-1, 2-dihydropyrazol-3-one* (**14b**). Yellow crystals, 85% yield, mp 170–172 °C from dilute ethanol; IR (KBr) ν= 3,500 (NH), 1,670 (C=O), 1,468, 1,380 (NO_2_) cmˉ^1^; ^1^H-NMR (DMSO-d_6_): δ = 7.39 (d, 2H, *J* = 9.0 Hz, H-2´,6´), 8.15 (d, 2H, *J* = 9.0 Hz, H-3´,5´), 8.19 (s, 1H, H-5) 11.06 (bs, 1H, NH), 11.88 (bs, 1H, NH); ^13^C-NMR (DMSO-d_6_): δ = 102.74 (C-4), 124.03 (C-3´, 5´), 124.86 (C-2´, 6´), 129.33 (C-5), 141.0 (C-1´), 143.83 (C-4´), 159.18 (C=O); MS (EI, 70 eV): *m/z* = 205 (M^+^); Anal. Calcd. for C_9_H_7_N_3_O_3_ (205.17): C, 52.69; H, 3.44; N, 20.48. Found: C, 52.74; H, 3.55; N, 20.59 %.

*4-(4-Nitrophenyl)-2-(4-methoxyphenyl)-1,2-dihydropyrazol-3-one* (**14c**). Orange crystals, 85% yield, mp 176–178 °C from dilute ethanol; IR (KBr) ν = 3,300 (NH), 1,660 (C=O), 1,466, 1,374 (NO_2_) cmˉ^1^; ^1^H-NMR (CDCl_3_): δ = 3.84 (s, 3H, OCH_3_), 6.90 (d, 2H, *J* = 9.0 Hz, Ar-H), 7.10 (d, 2H, *J* = 9.0 Hz, Ar-H), 7.45 (s, 1H, H-5), 7.59 (d, 2H, *J* = 9.0 Hz, Ar-H), 8.15 (d, 2H, *J* = 9.0 Hz, Ar-H), 8.90 (s, 1H, NH); ^13^C-NMR (CDCl_3_): δ = 55.57 (OCH_3_), 100.01 (C-4), 115.04 (C-3´´, 5´´), 117.82 (C-2´´, 6´´), 123.33 (C-3´, 5´), 129.17 (C-2´, 6´), 133.62 (C-1´´), 145.40 (C-1´), 145.47 (C-4´), 146.11 (C-5), 156.33 (C-4´´), 168.45 (CO); MS (EI, 70 eV): *m/z* = 311 (M^+^); Anal. Calcd. for C_16_H_13_N_3_O_4_ (311.29): C, 61.73; H, 4.21; N, 13.50. Found: C, 61.94; H, 3.95; N, 13.88 %.

## 4. Conclusions

*β*-Enamino esters could be easily obtained by reaction of ethyl phenylacetate derivatives with DMFDMA or with triethylorthoformate and piperidine in the presence of DMF. *β*-enamino esters are versatile intermediates for the synthesis of pyrrolidinones and pyridinones.
